# Jujube Peel Pigment-Loaded Thermosensitive Hydrogel with In Vitro Pro-Apoptotic and Antibacterial Activities

**DOI:** 10.3390/gels12090788

**Published:** 2026-09-01

**Authors:** Pei Zhang, Qianqian Chen, Shichao Chen, Huixia Guo, Mengru Ma, Yuge Pu, Zhenchao Jiang, Hongxia Liu, Peiran Guo, Xusheng Zhao, Ying Zhang, Xueyi Yang

**Affiliations:** College of Life Sciences, Luoyang Normal University, Luoyang 471934, China

**Keywords:** jujube peel pigment, thermosensitive hydrogel, HeLa cells, mitochondrial apoptosis, antibacterial activity, F127

## Abstract

Cancer remains a major global health concern, driving the search for safe and effective bioactive compounds from natural sources. Jujube peel red pigment (JP), an anthocyanin-rich extract, has shown preliminary bioactivity, yet its antitumor potential and delivery challenges remain underexplored. This study systematically evaluated the in vitro antitumor activity of JP and developed a thermosensitive hydrogel-based local delivery system (JP-H) to overcome its rapid diffusion and poor retention. JP exhibited selective cytotoxicity against HeLa cervical cancer and B16 melanoma cells, with no obvious toxicity to normal L929 and RAW264.7 cells. In HeLa cells, JP exerted antitumor effects by initiating mitochondrial-dependent apoptosis accompanied by elevated expression of Bax and cleaved Caspase-9/-3 as well as decreased Bcl-2 level, and arrested cell cycle at the G1/S phase by regulating CCND1, CDK2, CDK4, PCNA, MYC and TP53. To enable localized delivery, JP was incorporated into an injectable chitosan/gelatin/F127 thermosensitive hydrogel (JP-H), which exhibited rapid sol-gel transition at physiological temperature, shear-thinning behavior, and a porous microstructure. JP-H not only sustained JP release but also significantly enhanced antibacterial activity against *E. coli* and *S. aureus* compared to free JP. Furthermore, JP-H markedly inhibited HeLa cell migration and induced superior apoptotic/necrotic cell death in co-culture assays, outperforming free JP. Collectively, this work establishes JP as a multi-target antitumor agent and demonstrates JP-H as a promising local therapeutic platform combining sustained delivery, antibacterial protection, and enhanced anticancer efficacy for cervical cancer treatment.

## 1. Introduction

Cancer remains a major global health challenge. China, in particular, faces a disproportionate cancer burden relative to its population size, alongside a significant shift in the cancer spectrum [1]. Cervical cancer, ranking fourth in incidence among female malignant tumors worldwide and with an increasing incidence among younger individuals, presents a particularly critical challenge [2,3]. The development and progression of cervical cancer are associated with unchecked cell proliferation due to impaired regulation of the cervical cell cycle [4,5]. The transition of cells from the G1 to S phase represents a critical checkpoint in the cell cycle, primarily driven by the Cyclin D1/CDK4/6 complex and regulated by molecules such as proliferating cell nuclear antigen (PCNA), MYC proto-oncogene (MYC), and tumor protein p53 (TP53) [6,7,8,9,10,11]. Dysregulation of this pathway drives tumor proliferation, making it a key therapeutic target. Although conventional chemotherapeutics have demonstrated clinical efficacy, their utility is limited by significant toxicity, drug resistance, and poor local tumor control. Consequently, research efforts are increasingly directed toward safer naturally derived compounds and more efficient delivery systems [12].

Anthocyanins are a class of naturally occurring polyphenolic bioactive compounds widely found in plants [13]. They are derivatives of flavonoids and exhibit rich chemical structural diversity, primarily comprising various monomers such as cyanidin, delphinidin, and malvidin. Differences in the number of hydroxyl groups, as well as methylation and glycosylation modifications among these monomers, endow them with a broad range of biological activities and physicochemical properties [14]. Pharmacological studies have verified that anthocyanins exhibit prominent anti-tumor, antioxidant and anti-inflammatory activities [15]. Their anti-tumor mechanisms involve multi-target regulation: they exert potential therapeutic effects in the prevention and treatment of various cancers including cervical carcinoma, hepatocellular carcinoma and breast carcinoma by inhibiting tumor cell proliferation, inducing cell apoptosis and arresting the cell cycle progression [16,17]. Compared with conventional chemotherapeutic agents, anthocyanins feature natural sources, favorable biocompatibility and low systemic toxicity. In addition, they possess multiple pharmacological activities, thus showing great potential to be developed as adjuvant anti-tumor drugs.

However, the clinical application of anthocyanins is confronted with numerous bottlenecks. In terms of stability, the conjugated double bonds within their molecular structures are susceptible to light irradiation, temperature, pH values and oxidative surroundings, which easily trigger degradation or isomerization, drastically diminishing their bioactivity and imposing great challenges on their storage and practical application [18,19]. In terms of bioavailability, orally administered anthocyanins are prone to degradation by digestive enzymes in the gastrointestinal tract. After entering the liver via the portal vein, they undergo the first-pass effect. Furthermore, free-form anthocyanins are rapidly eliminated during systemic circulation, making it difficult to sustain therapeutically effective concentrations at tumor lesions [20,21]. Consequently, the anti-tumor efficacy of systemic administration is markedly compromised. In addition, although anthocyanins possess higher water solubility than some other natural bioactive substances, administration of free anthocyanins alone suffers from poor local retention and short action duration. Such drawbacks fail to meet the requirements for long-term localized intervention against tumors, which constitutes a critical factor restricting their clinical application.

Compared with systemic administration, local delivery strategies can precisely target tumor lesions, markedly elevate local drug concentration, reduce systemic drug exposure and alleviate toxic side effects simultaneously [22]. Hence, such approaches represent an optimal option for antitumor drug delivery. As a classic carrier for local drug delivery, hydrogels possess a three-dimensional porous network structure capable of encapsulating bioactive agents and realizing controlled drug release. In particular, thermosensitive hydrogels combine convenient injectability with in situ gelation properties: they exist as free-flowing liquids prior to administration, which can be precisely delivered to lesion sites via minimally invasive injection [23,24]. Once inside the body, they rapidly transform into solid gels under physiological temperature, effectively prolonging the local retention duration of loaded drugs and achieving long-term sustained release. Meanwhile, hydrogel carriers can improve the stability of anthocyanins and shield their bioactivity from adverse external environmental factors. In addition, hydrogels exhibit favorable biocompatibility, biodegradability and negligible immunogenicity [25]. They are highly suitable for the local delivery of anthocyanins and offer a feasible strategy to break through the bottlenecks restricting the clinical application of anthocyanins.

**Figure 1:**
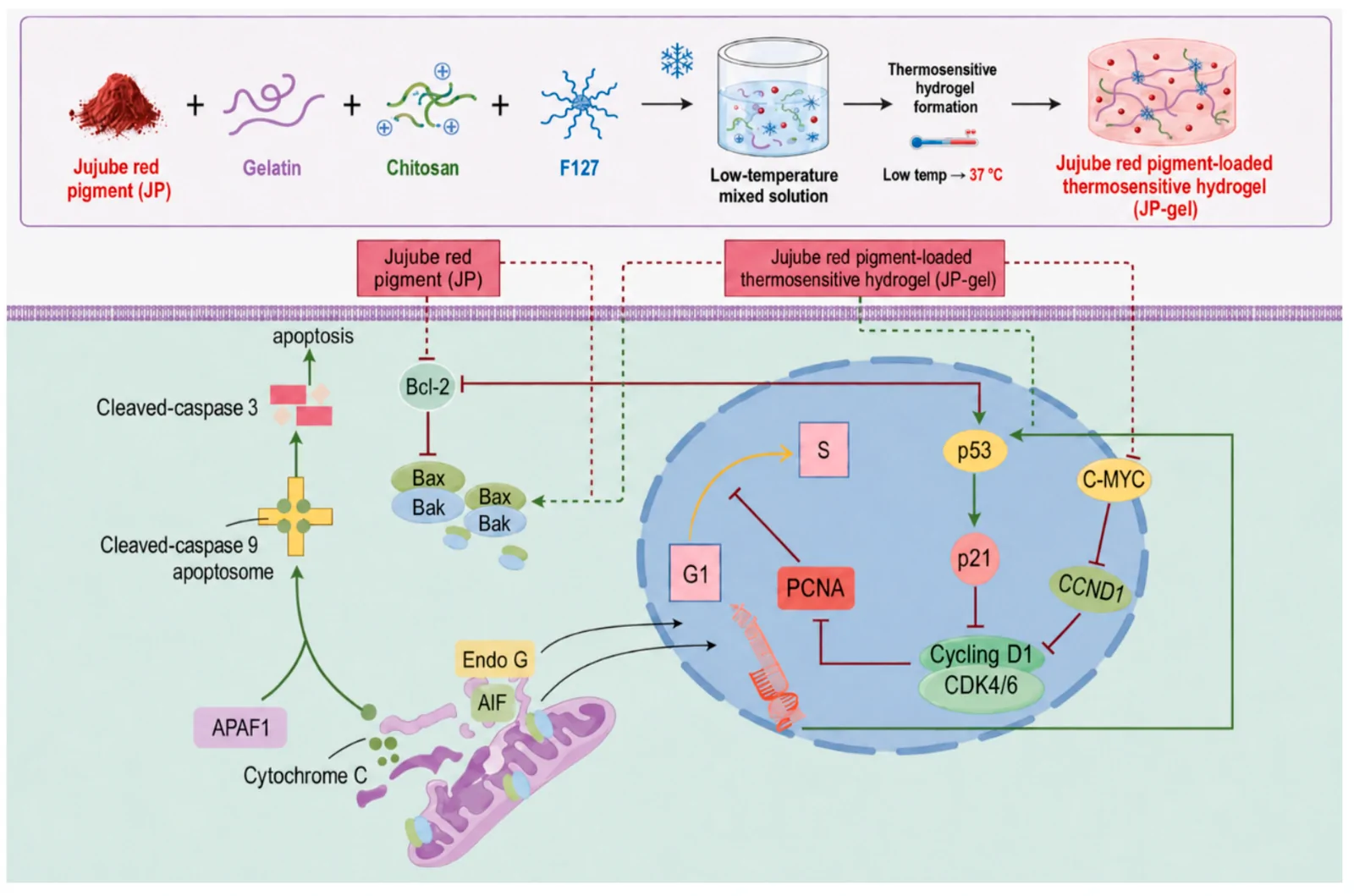
Schematic diagram of the preparation process of JP gel and the molecular mechanisms of antitumor action by free JP and JP gel.

Jujube (*Ziziphus jujuba Mill.*), a traditional Chinese medicinal and edible plant, is rich in various bioactive components. Jujube peel red pigment (JP), its main water-soluble anthocyanin component, possesses excellent antioxidant activity and certain physicochemical stability [26]. Preliminary studies have confirmed that JP can inhibit tumor cell proliferation and migration [27,28]. However, the antitumor activity of JP against cervical cancer cells has not been systematically evaluated, and it remains unclear whether it exerts its effects by modulating key cell cycle regulators. Moreover, the limitations of free JP in terms of stability and local retention still need to be addressed through delivery system optimization. Based on this, the present study first investigated the effects of JP on normal and tumor cells, focusing on its molecular mechanisms against HeLa cells. Subsequently, a chitosan/gelatin/F127 thermosensitive hydrogel local delivery system (JP-H) was constructed to achieve sustained local delivery of JP, and its antitumor efficacy was systematically evaluated (Figure 1). This work provides experimental evidence supporting the potential of JP as a natural-product-based drug candidate for cervical cancer, along with a corresponding delivery system.

## 2. Results and Discussion

### 2.1. Preparation Process and Component Analysis of JP

**Figure 2:**
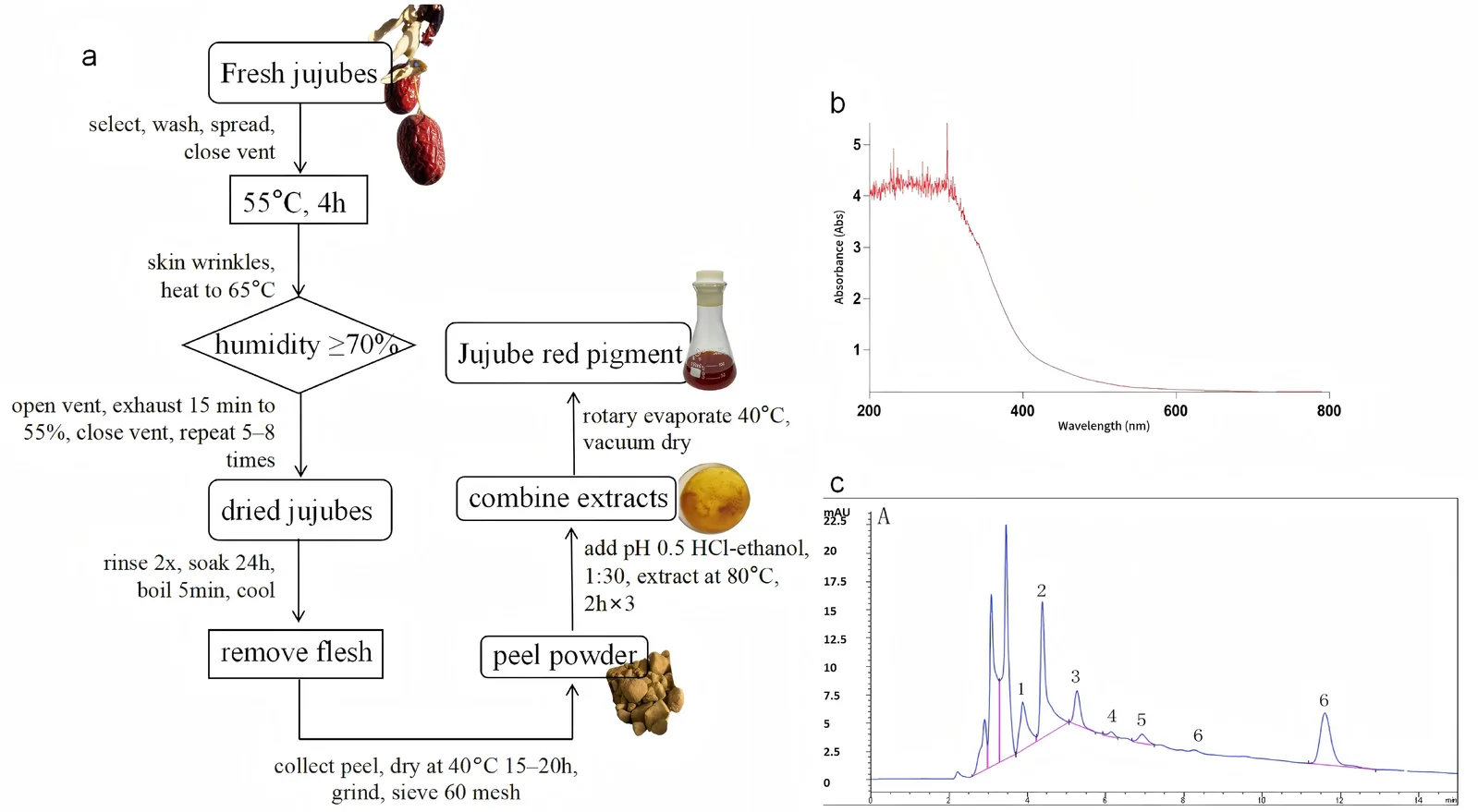
Preparation and characterization of JP: (**a**) preparation of JP; (**b**) UV-vis absorption spectrum of JP; (**c**) HPLC chromatogram of the extracted JP.

The binding between anthocyanins and plant tissues can be effectively disrupted by exposure to an acidified ethanol system, where thermal extraction was carried out at 80 °C to facilitate pigment dissolution (Figure 2a). JP exhibited its maximum absorption peak at 300 nm, with a strong and broad ultraviolet absorption band in the range of 200–350 nm, which is typical of anthocyanin compounds. Due to its conjugated molecular framework, JP exhibits pronounced ultraviolet light absorption, a characteristic that may underlie its antioxidant and photoprotective functions.

A distinct peak at a retention time of approximately 4.474 min was observed during HPLC analysis (Figure 2c), which was consistent with that of the authentic delphinidin-3-O-rutinoside standard (retention time: 4.45 min, Supplementary Figure S1) under identical chromatographic conditions. The identity of this component was further corroborated by its characteristic UV-vis absorption profile, which exhibited maximum absorption wavelengths at approximately 278 nm, in good agreement with previously reported values for anthocyanins [29]. Collectively, this peak was unequivocally identified as delphinidin-3-O-rutinoside. It is worth noting that in our previous work, the same extraction scheme was used for confirmed LC-MS/MS analysis, which further supported this result. The remaining low-intensity miscellaneous peaks at 3.943 min and 5.140 min indicated the presence of small amounts of polar impurities. The mutually validated results of UV-vis spectroscopy and HPLC demonstrated that delphinidin-3-O-rutinoside is the main constituent of JP, which also confers its characteristic red color and antioxidant activity.

### 2.2. In Vitro Cytotoxicity and Anti-Migration Activity of JP

**Figure 3:**
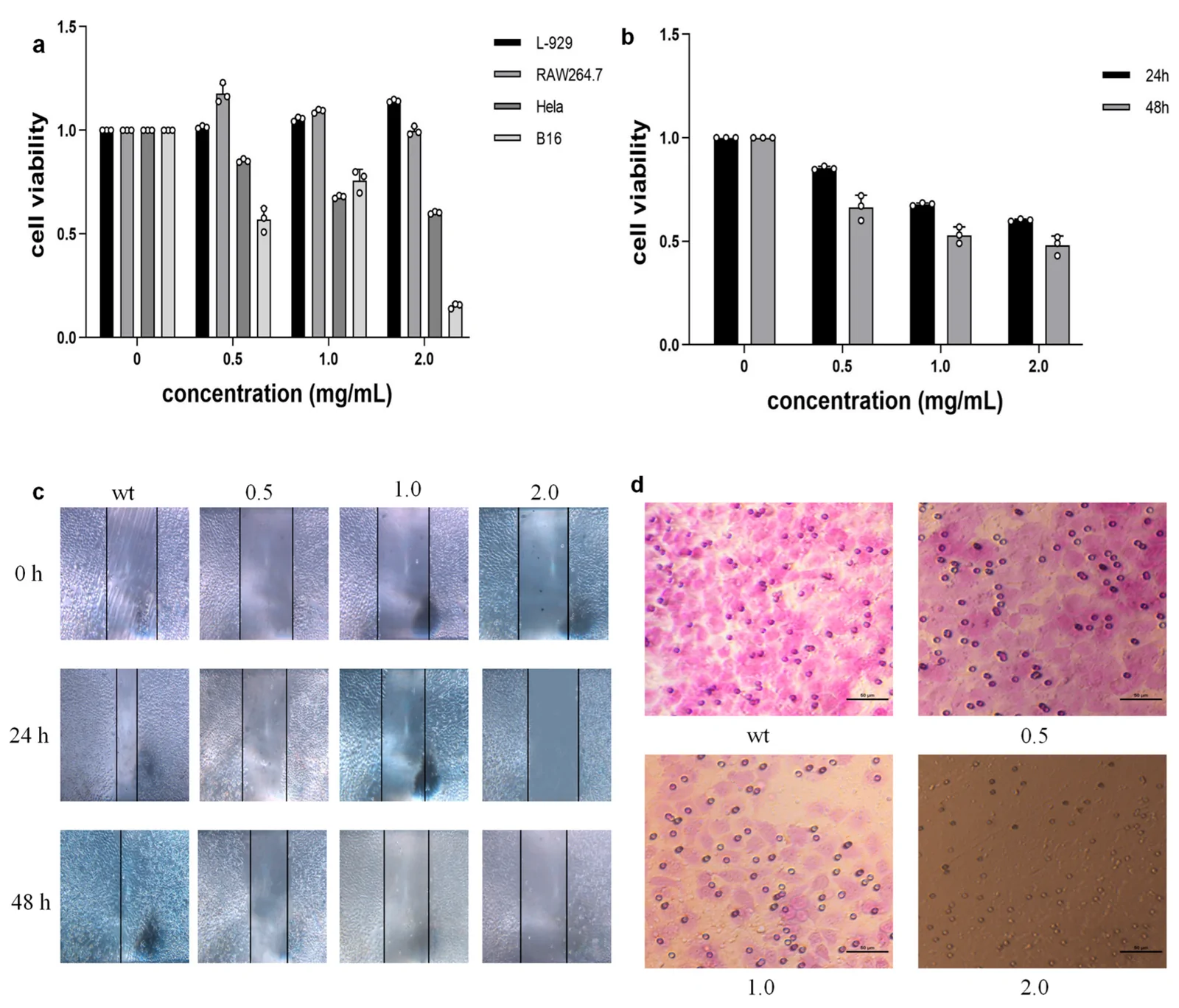
Cell viability assessment of JP on multiple cell lines and anti-migration performance of JP in HeLa cervical cancer cells: (**a**) effects of different concentrations of JP (0–2.0 mg/mL) treatment for 24 h on the viability of L929, RAW264.7, HeLa, and B16 cells; (**b**) effects of different concentrations of JP (0–2.0 mg/mL) treatment for 12 h/48 h on the viability of HeLa cells; (**c**) wound healing assay showing the migration of HeLa cells following JP treatment at 24 h and 48 h; (**d**) Transwell migration assay quantifying the number of migrated cells under different JP concentrations. Scar bars represent 50 µm.

JP showed distinct effects on two normal cell lines (Figure 3a). The viability of L929 cells increased slightly with rising JP concentration, whereas that of RAW264.7 cells decreased first and then increased, suggesting an adaptive or defensive cellular response to JP components. JP exhibited no obvious cytotoxicity within a certain dose range, possibly due to the natural origin of anthocyanins, which minimally impact normal cells [30]. In cancer cells, JP inhibited HeLa and B16 proliferation in a concentration-dependent manner, possibly due to oxidative stress and MAPK/PI3K pathways. The effects were slightly stronger on B16 cells than on HeLa. Moreover, JP inhibited HeLa cells in a concentration- and time-dependent manner (Figure 3b), consistent with typical dose-response patterns. High concentrations may induce ROS and mitochondrial damage, while cell cycle arrest or apoptosis are the predominant effects resulting from exposure to low doses [31]. However, these proposed mechanisms require further experimental validation.

Scratch wound healing and Transwell assays were performed to investigate the effect of JP on the migration ability of HeLa cells. In the scratch assay (Figure 3c), JP inhibited migration and wound closure in a concentration-dependent manner at 24 h, with marked suppression at 1.0 mg/mL and virtually no migration at 2.0 mg/mL. At 48 h, the control wounds exhibited nearly complete healing, whereas in the groups treated with 1.0 and 2.0 mg/mL of JP, the scratch remained largely unclosed, confirming sustained inhibition. Transwell assay results (Figure 3d) were also comparable: JP reduced the number of migrated cells dose-dependently, with the strongest effect observed at 2.0 mg/mL. The data demonstrate that JP effectively inhibits HeLa cell migration in a dose-dependent manner.

Tumor metastasis is the primary cause of cancer treatment failure [32]. JP significantly inhibited both the horizontal and vertical migration abilities of HeLa cells in a concentration-dependent fashion. Often, the inhibition of cell migration ability is associated with impaired epithelial-mesenchymal transition (EMT) progression or reduced activity of matrix metalloproteinases (MMPs) [33,34]. MYC serves as a crucial regulator of EMT [35]. While this study did not directly examine the expression of EMT markers or MMPs, the significant suppression of MYC by JP suggests that JP inhibits migration by interfering with the EMT process. This provides a direction for subsequent research to further explore its anti-metastatic mechanism.

### 2.3. JP Induces Apoptosis in HeLa Cells and Its Molecular Mechanism

**Figure 4:**
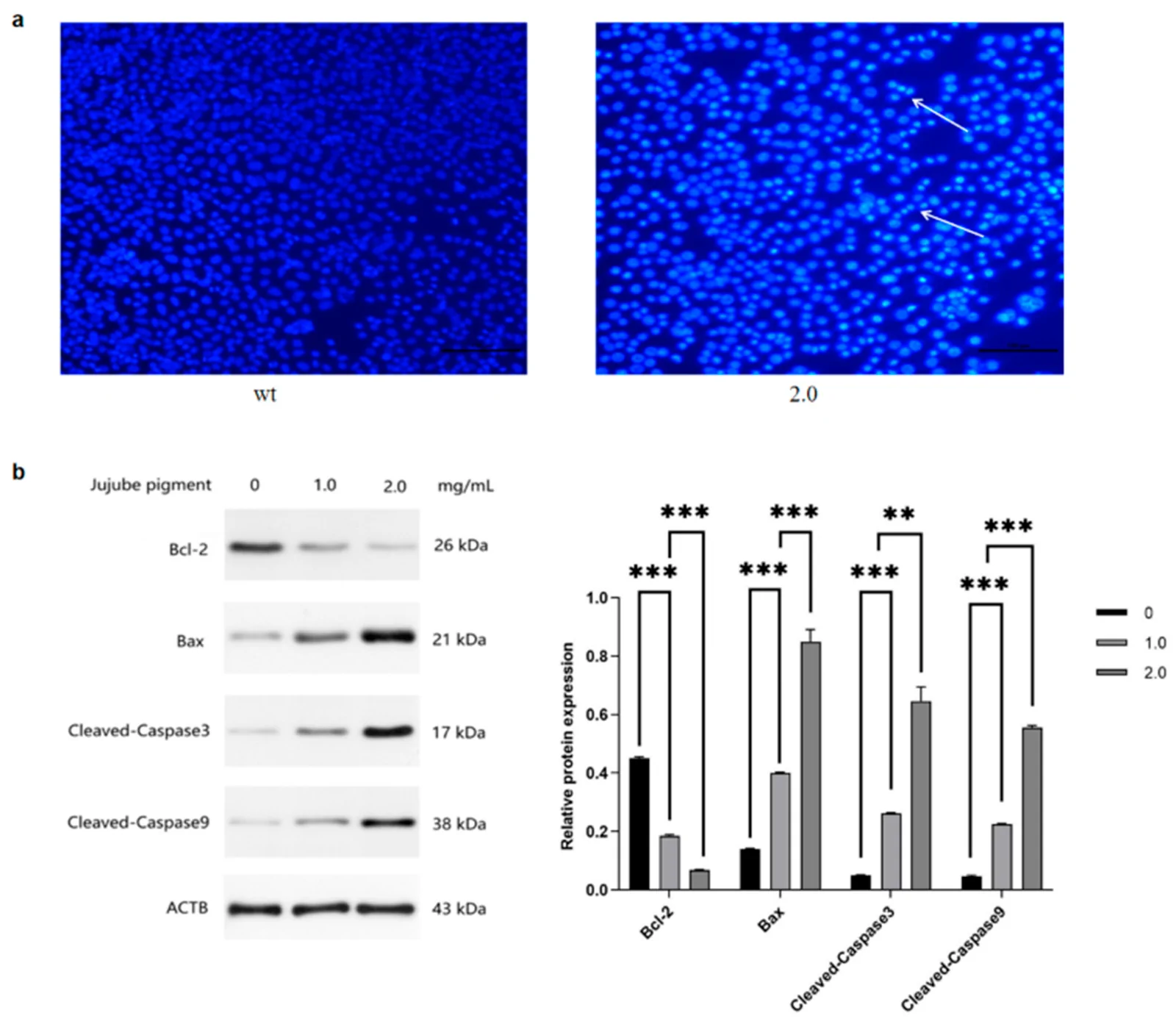
JP induces apoptosis in HeLa cells and regulates Bax and Bcl-2 expression: (**a**) representative images of Hoechst 33258 staining showing apoptotic nuclear morphology in HeLa cells treated with 0 mg/mL or 2.0 mg/mL JP; (**b**) Western blot analysis of Bax and Bcl-2 expression in HeLa cells after JP treatment. Data presented as the mean ± SD, *n* = 3. Statistically significant ** *p* < 0.01, *** *p* < 0.01. White arrows indicate apoptotic cells characterized by condensed or fragmented nuclei (Hoechst).

In addition to migration inhibition, tumor cell apoptosis is a key mechanism of action of anticancer drugs. Hoechst 33258 staining and Western blot were carried out to investigate JP-induced apoptosis in HeLa cells (Figure 4). As shown by the staining results (Figure 4a), the cells exhibited typical apoptotic nuclear features after 48 h treatment with 2.0 mg/mL JP, including chromatin condensation, enhanced fluorescence, and nuclear fragmentation. However, the control nuclei remained intact. Western blot (Figure 4b) showed a JP-induced dose-dependent downregulation of anti-apoptotic Bcl-2 and upregulation of pro-apoptotic Bax, resulting in a significant increase in the Bax/Bcl-2 ratio. Cleavage of Caspase-9 and Caspase-3 was observed. This Bcl-2/Bax imbalance serves as the core switch of the mitochondrial apoptotic pathway and induces programmed cell death by promoting mitochondrial outer membrane permeabilization and subsequent activation of the Caspase-9/-3 cascade.

This shift in the Bax/Bcl-2 ratio leads to increased mitochondrial outer membrane permeability, the release of cytochrome c [36]. These findings are consistent with the reported mechanism of apoptosis induction through the regulation of Bcl-2 family proteins by various natural products [37]. The results of this study show that the cleaved forms of Caspase-9 and Caspase-3 are up-regulated by JP and indicate that it activates the caspase cascade through the intrinsic mitochondrial apoptotic pathway. Activation of this pathway may contribute to greater tumor selectivity than that achieved by conventional cytotoxic agents, although this hypothesis requires further experimental validation. However, the fact that JP may also regulate other apoptosis-related pathways such as endoplasmic reticulum stress- or death receptor-mediated apoptosis cannot be ruled out and requires further mechanistic investigation.

### 2.4. JP Regulates the Expression of Cell Cycle-Related Genes

**Figure 5:**
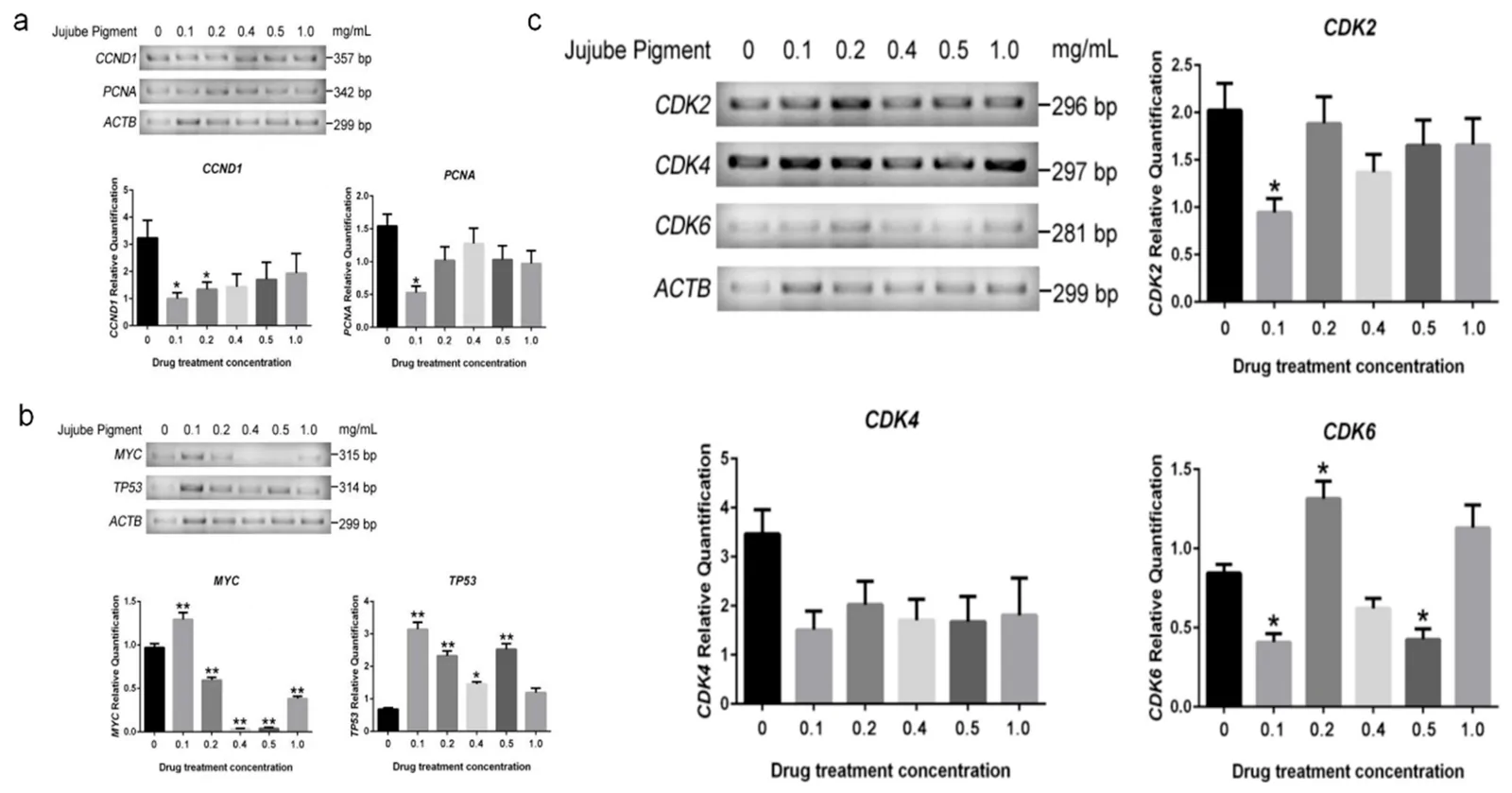
JP modulates the cell cycle regulatory network in HeLa cells: (**a**) qPCR analysis of key cell cycle regulators (CCND1, MYC, PCNA, and TP53); (**b**) qPCR analysis of CCND1, MYC, PCNA, and TP53; (**c**) qPCR analysis of CDK2, CDK4, and CDK6. Data are presented as the mean ± SD, *n* = 3. * *p* < 0.05, ** *p* < 0.01.

In an attempt to probe the molecular mechanism of JP-induced HeLa cell growth inhibition, the expression of cell cycle-related genes was examined (Figure 5a,b). The pro-proliferative genes CCND1, PCNA [38,39], and MYC were downregulated following JP treatment (except for MYC upregulation at 0.1 mg/mL), whereas the tumor suppressor TP53 was upregulated at low concentration (0.1 mg/mL). However, the effect diminished at higher doses. These transcriptional changes collectively suggest a coordinated suppression of cell cycle progression, with the downregulation of CCND1, PCNA, MYC, CDK2, and CDK4, and the upregulation of TP53, pointing toward a potential G1/S phase blockade.

To further assess cell cycle execution, the expression of CDKs was analyzed (Figure 5c). Across most concentrations, JP consistently suppressed CDK2 and CDK4, with only slight attenuation observed at 0.3 mg/mL. CDK6, on the contrary, exhibited a non-monotonic response with downregulation at 0.1 mg/mL followed by upregulation at 0.3 mg/mL, and subsequent re-inhibition at 0.4 mg/mL, suggesting a complex, subtype-selective regulation effect of JP on CDKs which may in turn be attributed to possible feedback or compensatory mechanisms among CDK family members.

A key characteristic of malignant tumor proliferation is uncontrolled cell cycle progression [4]. Following the establishment of JP’s pro-apoptotic effect, the study further investigated its cell cycle arrest activity. JP significantly downregulated the expression of CCND1, MYC, and the proliferation marker PCNA. The expression of the tumor suppressor gene TP53 was, however, upregulated. CCND1 complexation with CDK4/6 is the key factor driving cells through the G1 checkpoint; therefore, its downregulation can result directly in G1 phase arrest. The inhibition of MYC, which functions as a global transcriptional regulator, further weakens the cell proliferation signaling network [40]. However, JP exhibited differential regulation of CDK family members, inhibiting CDK2 and CDK4, while showing a concentration-dependent bidirectional regulation of CDK6.

This differential sensitivity observed within the CDK family may originate from a complex regulatory pathway controlling progression through the cell cycle, with individual members of the CDK family having specific functional roles, compensatory pathways, and feedback from other pathway components. Further work is needed to delineate the molecular mechanisms behind this selective regulation. In summary, the transcriptional changes caused by the treatment reflect a coordinated mechanism of cell cycle progression suppression, which is partially consistent with previous reports regarding polyphenols, such as curcumin and quercetin, exerting anti-tumor effects through similar pathways [41,42]. The atypical regulatory pattern exhibited by CDK6 in response to JP indicates that its mechanism of action may involve novel cellular targets.

Compared with natural anti-tumor compounds such as Moscatilin and Ma [43,44], JP demonstrates potential for multi-target and multi-pathway synergistic effects. It inhibits tumor growth by regulating classical cell cycle checkpoints and apoptotic switches and also suppresses the migratory behavior of cancer cells. This multi-faceted intervention characteristic reduces the risk of drug resistance associated with single-target drugs [45]. However, the exact primary target, including whether it directly interacts with a specific kinase or receptor, remains unclear and is a key issue that needs to be addressed in future research.

### 2.5. Physical and Chemical Characteristics of JP-H

**Figure 6:**
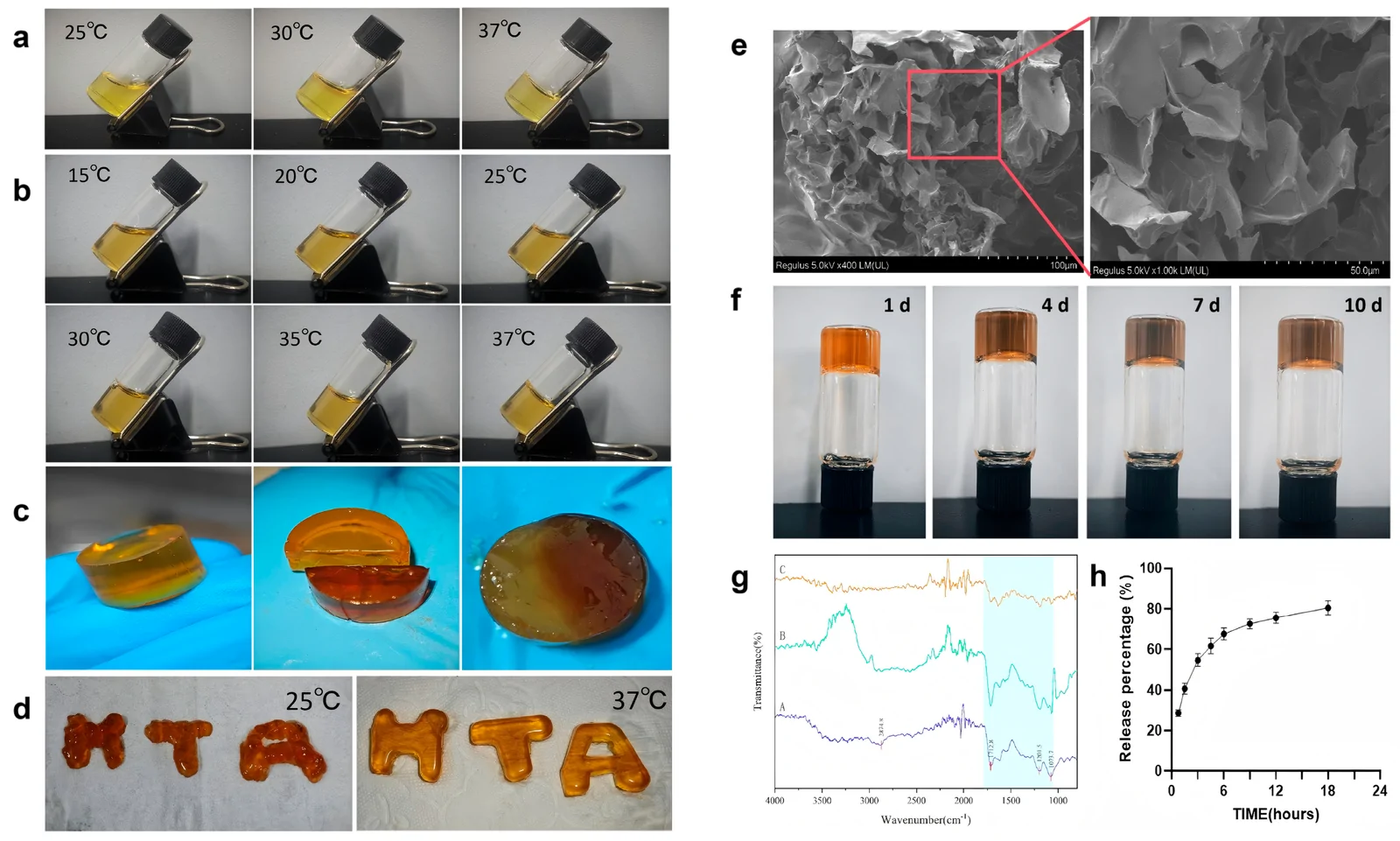
Thermoresponsive sol-gel transition, macroscopic morphology, self-healing performance, microstructure and chemical characterization of JP-H: (**a**) digital photographs illustrating the temperature-dependent sol-gel transition behavior of blank hydrogel at 20 °C, 30 °C and 37 °C; (**b**) digital photographs showing the temperature-dependent sol-gel behavior of JP-H precursor solution at 15, 20, 25, 30, 35 and 37 °C; (**c**) macroscopic photographs of the as-prepared JP-H, including intact hydrogel, cut cross-section, and self-healing performance after fracture and reconnection; (**d**) injectable and moldable performance of JP-H precursor: the solution was cast into letter-shaped molds at 25 °C (sol state) and transformed into solid hydrogel at 37 °C; (**e**) SEM images displaying the porous internal microstructure of lyophilized JP-H with different magnifications, the red square represents that the area in the right picture and the left picture is the same field of vision; (**f**) macroscopic inversion observation of JP-H hydrogel incubated at 37 °C for 1, 4, 7 and 10 days; (**g**) FT-IR spectra of (A) free JP, (B) JP-H composite hydrogel, and (C) blank hydrogel matrix; (**h**) in vitro cumulative release percentage of JP from the thermosensitive hydrogel.

To address the bottlenecks of rapid in vivo diffusion and poor local retention of free JP, this study constructed a gelatin-chitosan-based thermosensitive hydrogel as an injectable sustained-release depot for JP. This system establishes a reinforced three-dimensional network through hydrogen bonding and electrostatic interactions [34], while the chitosan component possesses anti-inflammatory and antibacterial activities, synergizing with JP to enhance its antitumor efficacy. The thermosensitivity imparted by F127 ensures both a sufficient operational window for injection and rapid in situ gelation post-administration, while guaranteeing a high encapsulation efficiency of JP [46,47]. Inversion vial tests confirmed that both the drug-free and JP-loaded hydrogels remained in a sol state at 20 °C and 30 °C, but underwent responsive solidification at 37 °C, indicating that JP loading did not compromise the inherent thermosensitive properties of the hydrogel (Figure 6a,b). It should be noted that the thermoresponsive properties of JP-H were evaluated under idealized laboratory conditions, whereas physiological environments or biological buffer systems may significantly alter its thermal behavior. Shymborska systematically investigated the impact of various pH buffer solutions on the temperature-responsive properties of POEGMA brush coatings [48]. They found that, compared with pure water, acidic and neutral/alkaline buffer solutions reduced the LCST from 22 °C to 9–11 °C and 16–17 °C, respectively, and attenuated the extent of temperature-induced wettability changes.

Their study demonstrated that buffer ions (e.g., Na^+^ and phosphate/citrate anions) can disrupt polymer hydration layers or associate with glycol groups, thereby interfering with polymer-water interactions and altering phase transition behavior. Although the F127/chitosan/gelatin matrix used in our study differs chemically from POEGMA, these findings suggest that when JP-H is applied in buffer-containing physiological environments (e.g., body fluids and cell culture media), its actual gelation temperature, release kinetics, and antitumor activity may deviate from the current in vitro results. Therefore, future studies should systematically evaluate the thermoresponsive stability of JP-H in more physiologically relevant buffer systems (e.g., PBS and cell culture media) to ensure its predictability and efficacy for in vivo applications. Additionally, the formulation possesses shear-thinning properties and self-healing capacity, which facilitate facile injection and allow the gel network to rapidly reassemble and recover its structural integrity post-injection (Figure 6c). The injectable precursor solution readily conformed to molds with intricate letter-shaped geometries, illustrating its excellent moldability and adaptability to defects with complex and irregular architectures (Figure 6d).

Such abundant porous architecture provides sufficient space for cell adhesion and proliferation, and also creates transport channels for sustained long-term release of loaded JP pigment, laying a structural foundation for the prolonged antibacterial and antitumor effects of JP-H (Figure 6e). After incubation at 37 °C for 1, 4, 7 and 10 days, the hydrogel maintained intact morphology without flow or collapse upon inversion, demonstrating that the crosslinked network can sustain stable gel state for an extended period, which is conducive to local retention at lesion sites (Figure 6f). FT-IR analysis (Figure 6g) was performed to confirm the successful incorporation of JP into the hydrogel network and to investigate intermolecular interactions between the pigment and the polymer matrix. The spectrum of the blank hydrogel (C) exhibited characteristic absorption bands of the chitosan/gelatin/F127 composite: a broad O-H/N-H stretching vibration at ~3400 cm^−1^, C-H stretching at ~2920 cm^−1^, amide I (C=O stretching) at ~1640 cm^−1^, and amide II (N-H bending) at ~1550 cm^−1^, consistent with the polysaccharide and protein components. The free JP spectrum (A) displayed typical signals for polyphenolic and glycosidic structures, including O-H stretching (~3400 cm^−1^), aromatic C=C ring vibrations (~1600 and 1520 cm^−1^), and C-O-C glycosidic linkages (~1070 cm^−1^). In the JP-H composite spectrum (B), all major characteristic peaks of both the hydrogel matrix and JP were preserved without the appearance of new bands, indicating that the chemical integrity of each component was maintained during formulation.

Notably, the O-H stretching band in JP-H exhibited a slight broadening and a minor redshift compared to that of the blank hydrogel, while the aromatic C=C peaks of JP showed subtle shifts from 1600 to 1606 cm^−1^. These spectral changes suggest the formation of intermolecular hydrogen bonds between the hydroxyl groups of JP’s polyphenolic rings and the amino/hydroxyl groups of chitosan and gelatin. Importantly, the absence of new covalent bonds (e.g., ester or Schiff-base linkages) confirms that JP was physically encapsulated within the hydrogel matrix rather than chemically conjugated. This stable physical entrapment is favorable for sustained release and ensures that the bioactivity of JP is not compromised by chemical modification. The encapsulation efficiency of JP in the thermosensitive hydrogel was determined to be 86.3% ± 2.1%, and the drug loading was 8.6% ± 0.3% (*n* = 3), indicating efficient entrapment via physical interactions. The in vitro release profile exhibited a biphasic pattern: an initial burst release of approximately 45% within the first 3 h, followed by sustained release over 20 h, reaching a cumulative release of 80% (Figure 6h).

### 2.6. Antibacterial Performance of JP-H

**Figure 7:**
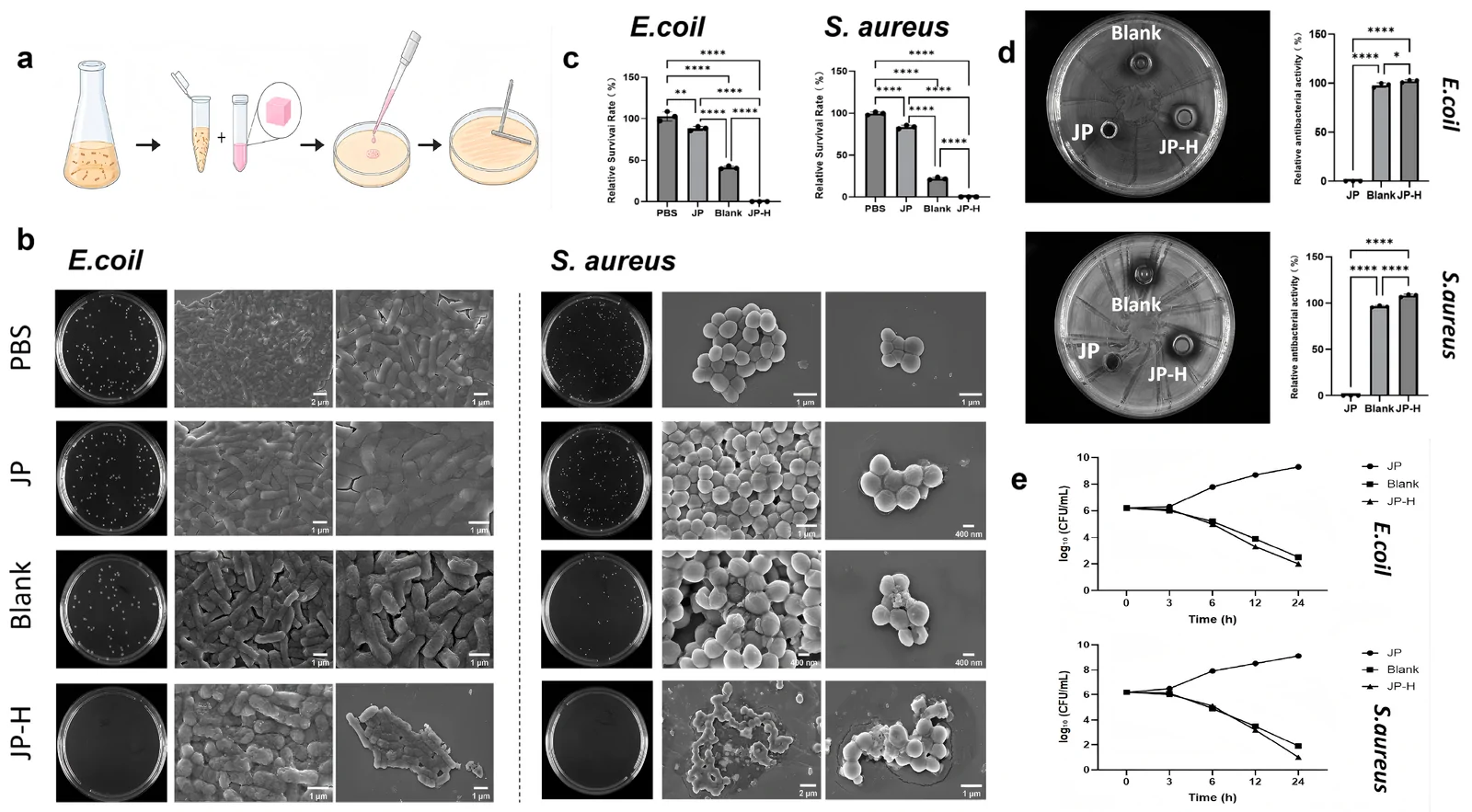
Antibacterial activity of free JP and JP-H against *Escherichia coli* and *Staphylococcus aureus*. (**a**) Schematic illustration of the antibacterial assay. (**b**) Representative colony-counting images and SEM micrographs of *E. coli* and *S. aureus* following treatment with PBS, free JP, blank hydrogel, or JP-H. (**c**) Quantitative analysis of relative bacterial survival determined by colony counting. (**d**) Representative disk-diffusion images and quantitative analysis of inhibition-zone diameters for free JP, blank hydrogel, and JP-H. (**e**) Time-kill curves for the antibacterial activity of the JP-loaded hydrogel, free JP, and blank hydrogel. Data are presented as the mean ± SD, *n* = 6. Statistically significant * *p* < 0.05, ** *p* < 0.01, **** *p *< 0.0001. Different symbols indicate statistically significant differences as defined in the figure.

The antibacterial activity of free JP, the blank hydrogel, and JP-H was evaluated against the Gram-negative bacterium *Escherichia coli* and the Gram-positive bacterium *Staphylococcus aureus* using colony-counting, disk-diffusion, and SEM analyses (Figure 7). Abundant colonies were observed in the PBS control group, indicating unrestricted bacterial growth. Free JP moderately reduced bacterial survival, confirming that the pigment extract possesses intrinsic antibacterial activity. The blank hydrogel also decreased bacterial survival to some extent, which may be associated with the membrane-active properties of protonated amino groups in chitosan. Among all treatments, JP-H produced the lowest bacterial survival rates for both strains, with significantly stronger antibacterial effects than free JP and the blank hydrogel.

The disk-diffusion results were generally consistent with the colony-counting data. JP-H generated the most pronounced inhibition zones against both *E. coli* and *S. aureus*, whereas free JP produced smaller inhibition zones and the blank hydrogel showed little or limited diffusion-mediated inhibition. The difference between the colony-counting and disk-diffusion results for the blank hydrogel may reflect the contact-dependent antibacterial activity of chitosan and its limited diffusion through the agar matrix. By contrast, the inhibition zones surrounding JP-H indicate that antibacterial constituents of JP were able to diffuse from the hydrogel into the surrounding medium.

SEM further revealed treatment-dependent alterations in bacterial morphology. Bacteria in the PBS group retained intact cellular contours and relatively smooth surfaces. The blank hydrogel and free JP caused varying degrees of surface roughening, wrinkling, and deformation. More extensive structural damage was observed after JP-H treatment. *E. coli* displayed pronounced shrinkage, collapse, and disruption of the rod-shaped cellular envelope, whereas *S. aureus* exhibited surface deformation, aggregation, and loss of its regular spherical morphology. These morphological changes are consistent with compromised bacterial envelope integrity. Collectively, the results demonstrate that incorporation of JP into the thermosensitive chitosan/gelatin/F127 hydrogel produces stronger antibacterial activity than either free JP or the unloaded hydrogel under the tested conditions. This enhancement may result from the combined antibacterial effects of JP-derived polyphenolic components and the chitosan-containing matrix, together with improved local retention and contact between JP and bacterial cells.

However, because the membrane permeability of JP was not directly assessed, the enhanced antibacterial activity cannot be conclusively attributed to a specific membrane-disruption mechanism. To further evaluate the time-dependent antibacterial efficacy of JP-H, time-kill curve assays were performed against *E. coli* and *S. aureus* (Figure 7e). JP-H exhibited the most potent and rapid antibacterial activity against both strains, achieving a > 3-log_10_ reduction in viable bacterial counts within 6 h, and complete bacterial killing (≥ 5-log_10_ reduction) within 12 h. In contrast, free JP and blank hydrogel showed only moderate bactericidal effects, with approximately 1.5- and 1.0-log_10_ reductions, respectively, at the same time points. These time-kill data are fully consistent with the colony-counting and disk-diffusion results, further confirming that the sustained release of JP from the thermosensitive hydrogel significantly enhances its antibacterial efficacy in a time-dependent manner.

### 2.7. Analysis of Migration Inhibition and Cytotoxicity of JP-H Against HeLa Cells

**Figure 8:**
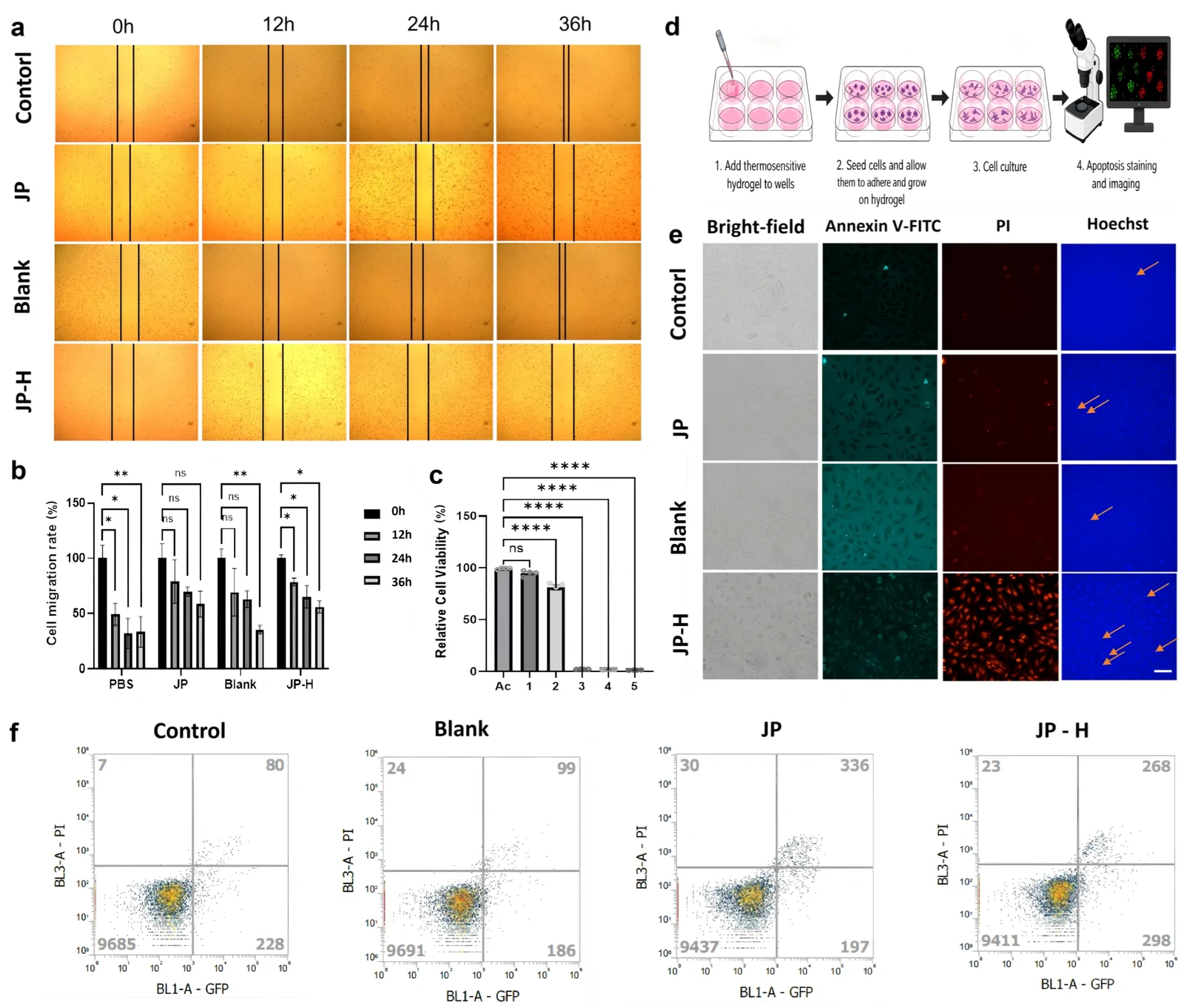
Anti-migration and pro-apoptotic effects of free JP and JP-H on HeLa tumor cells: (**a**) bright-field scratch wound images of HeLa cells treated with Control, free JP, blank hydrogel, and JP-H at 0 h, 12 h, 24 h and 36 h; (**b**) quantitative statistical analysis of cell migration rates corresponding to the scratch wound assay in panel (**a**); (**c**) relative viability of HeLa cells after treatment with serial concentrations of JP; (**d**) schematic workflow of the co-culture system of thermosensitive hydrogel and tumor cells, followed by multi-channel fluorescence apoptosis staining; (**e**) representative fluorescence images of cells stained with Annexin V-FITC (green), propidium iodide (PI, red), and Hoechst 33342 (blue). Bright-field images show the corresponding cellular morphology. Scale bar represent 100 μm. arrows indicate apoptotic cells characterized by condensed or fragmented nuclei (Hoechst staining); (**f**) flow cytometry scatter plots of Annexin V-FITC/PI double staining for quantitative detection of apoptosis ratios in HeLa cells from Control, Blank, free JP and JP-H groups. Data are presented as the mean ± SD, *n* = 6. * Statistically significant * *p* < 0.05, ** *p* < 0.01, **** *p *< 0.0001, ns indicates no significant difference.

The effects of JP-H on tumor cell migration and survival were evaluated using a wound healing assay and live/dead fluorescence staining in HeLa cells. PBS, blank hydrogel, and free JP were also evaluated in comparison. Following 24 h culture, both the PBS and Blank groups showed substantial closure of the scratch gaps (Figure 8a,b), with no significant difference in migration rates between the two groups. The free JP group demonstrated a slightly higher degree of wound closure in comparison to the controls, indicating that a low JP concentration did not inhibit HeLa cell migration, but rather slightly promoted it. In the JP-H group, the scratch width exhibited almost no change in comparison with the 0 h baseline, demonstrating a significant inhibition of cell migration. In the Live/dead cell staining assay (Figure 8c,e), the PBS and Blank groups displayed bright FITC green fluorescence (live cells) with only sparse PI red fluorescence (dead cells). The live cell fluorescence intensity in the free JP group was comparable to the control, with only a few PI-positive cells. The JP-H group, however, exhibited virtually complete quenching of FITC fluorescence, whereas extensive bright PI red fluorescence pointed towards severe necrosis and apoptosis of HeLa cells, which was consistent with the failure of cells to migrate into the scratch area as demonstrated by the scratch assay.

Quantitative flow cytometry analysis via Annexin V-FITC/PI double staining was adopted to precisely quantify the cellular apoptotic ratio (Figure 8f). The total apoptotic population of the Control and blank hydrogel groups remained at a low level, with only slight apoptosis triggered by free JP treatment. In contrast, JP-H treatment markedly elevated the proportion of early and late apoptotic HeLa cells. Collectively, these results illustrated that JP could simultaneously inhibit tumor cell migration and induce cellular apoptosis, and the thermosensitive hydrogel delivery system further amplified these two antitumor functions, highlighting the great potential of JP-H as a local therapeutic platform for cervical cancer treatment. The FT-IR and release behavior indicate that long-term sustained release of JP within the three-dimensional hydrogel network mediates the anticancer activity of the hydrogel. The diffusion and dilution of free JP rapidly in the culture medium maintains only a low bioactive concentration, with weak anticancer efficacy. JP-H, on the other hand, gels in situ and maintains a persistently high local concentration of JP in the cellular microenvironment. The disruption of cell membrane integrity and redox homeostasis by exposure to high-dose polyphenols induces apoptosis and necrosis, which is manifested as extensive PI-positive signals and complete inhibition of scratch healing. This is also consistent with the concentration-dependent biological response elicited by JP. JP-H performs dual roles as a broad-spectrum antibacterial and anti-cervical cancer agent, offering potential value for minimally invasive local treatment of cervical cancer with concomitant infection.

However, this study has several limitations. First, the lack of evaluation of biocompatibility with normal epithelial cells, the absence of toxicity assessment of high concentrations of polyphenols toward normal tissues, and the lack of validation in relevant in vivo animal models remain to be addressed for future preclinical development. In addition to these overall pharmacological considerations, mechanistic evidence at the cellular level is also incomplete. Specifically, direct flow cytometric cell cycle analysis was not performed, leaving the precise checkpoint targeted by JP yet to be definitively identified. Likewise, upstream mitochondrial events—including ROS generation and ΔΨm disruption—were not directly measured, which further limits the mechanistic interpretation of the observed antitumor effects. Furthermore, protein-level validation of key G1/S regulators (p21, p27, RB phosphorylation, E2F, Cyclin E, and CDK6) remains to be completed. Despite these mechanistic gaps, the current multi-layered evidence—apoptotic morphology (Hoechst 33258), Annexin V/PI flow cytometry, mitochondrial markers (Bax/Bcl-2 ratio, cleaved Caspase-9/-3), and coordinated transcriptional changes in cell cycle regulators (CCND1, CDK2, CDK4, PCNA, MYC, and TP53)—collectively supports a mechanistically coherent model in which JP simultaneously triggers mitochondrial apoptosis and interferes with G1/S phase progression. These unresolved issues, including the aforementioned cell cycle and mitochondrial analyses as well as protein-level validation, are planned as part of our ongoing investigations to fully elucidate the molecular pathway underlying JP’s antitumor activity in cervical cancer cells.

## 3. Conclusions

This study combines the antitumor potential of a natural-product jujube peel with an injectable thermosensitive hydrogel-based local delivery. JP extracted from jujube peel inhibited HeLa cell viability and migration, induced Bcl-2/Bax regulation and Caspase-9/-3 activation to trigger mitochondrial apoptosis, and altered the expression of key cell-cycle regulators, including CCND1, CDKs, PCNA, MYC, and TP53, interfering with G1/S phase progression. To overcome the limitations in the localized delivery of free JP, a JP-loaded chitosan/gelatin/F17 thermosensitive hydrogel was fabricated. With injectable delivery, temperature-responsive gelation, local retention potential, bactericidal potential against *E. coli* and *S. aureus*, and tumoricidal activity against HeLa cells, JP-H functions as a local antitumor delivery platform. The developed JP-loaded thermoresponsive hydrogel constitutes a promising localized therapeutic platform integrating the sustained drug retention of the hydrogel matrix with the antibacterial and antitumor properties of the naturally derived pigment JP. In future studies, flow cytometric cell cycle analysis will be performed to confirm G1/S phase arrest, and the antitumor mechanism of JP will be further elucidated through a combination of cellular assays and in vivo validation in animal models.

## 4. Materials and Methods

Extraction of red pigment from jujube peel: Fresh, washed gray jujubes were oven-dried first at 55 °C for 4 h and then at 65 °C, with intermittent venting to release moisture until the internal humidity reached 70%. The process was repeated 5–8 times. The dried jujubes were soaked in cold water for 24 h, boiled for 5 min, and cooled. Jujube Peels were separated, dried at 40 °C for 15–20 h, pulverized, and sieved through a 60-mesh sieve (Φ200, Xinxiang Sieve Co., Ltd., Xinxiang, China). The powder was extracted thrice with ethanolic hydrochloric acid solution (pH 0.5) at a solid-to-liquid ratio of 1:30 at 80 °C for 2 h each. Dried crude JP was obtained by combining and concentrating the extracts.

Spectroscopic and chromatographic characterization: The jujube red pigment solution (0.5 mg/mL) was scanned across the full wavelength range using a UV-Vis spectrophotometer (U-3900, Hitachi High-Tech, Tokyo, Japan) to determine its maximum absorption wavelength.. High performance liquid chromatography (HPLC) was also employed for analyzing the constituents of jujube peel red pigment under the following chromatographic conditions: injection volume 20 μL, chromatographic column Eclipse XDB-C18 column (1260 Infinity II, Agilent Technologies, Santa Clara, CA, USA), detection wavelength 280 nm, column temperature 30 °C, 10% formic acid aqueous solution as mobile phase A, a mixed solution of methanol, acetonitrile and 10% aqueous formic acid as mobile phase B was, and the ratio of A to B as 67:33 (*v*/*v*). Standard delphinidin-3-O-rutinoside and the prepared jujube peel red pigment sample were separately injected and analyzed. Each measurement was carried out in triplicate, and the retention time as well as peak areas were recorded. Standard delphinidin-3-O-rutinoside (purity ≥ 98%, CAS:15674-58-5) was purchased from Sigma-Aldrich (St. Louis, MO, USA). The standard was stored at −20 °C in the dark prior to use.

Cell lines: HeLa, B16, L929, and RAW264.7 cells, purchased from Huatuo Biotechnology (Shanghai, China), were cultured in Procell special media supplemented with 10% fetal bovine serum (FBS). The cells were maintained in a closed incubator (3111, Thermo Scientific, Waltham, MA, USA) at 37 °C in a 5% CO_2_ atmosphere. Cells were enzymatically dissociated using EDTA-trypsin and subcultured at 2-day intervals.

Cell viability assay: HeLa, B16, L929, and RAW264.7 cells in the logarithmic phase were trypsinized, resuspended, and seeded into 96-well plates, in a medium containing 5% serum, at a density of 9 × 10^3^ cells/well (100 μL/well). When cell confluence exceeded 80%, following a 24 h incubation at 37 °C with 5% CO_2_, the medium was discarded. Cells were then treated with JP at concentrations of 0.5, 1, and 2 mg/mL (100 μL/well, 5% serum) for 24 h, with three replicate wells per group. Subsequently, 10 μL MTT (5 mg/mL) was added to individual wells. Following 4 h incubation and subsequent removal of the supernatant, 110 μL DMSO was added to individual wells to dissolve formazan crystals. Absorbance was measured at 490 nm using a microplate reader (Synergy H1, BioTek, Winooski, VT, USA). Given that subsequent antitumor mechanism studies were primarily conducted using HeLa cells as the main model, an additional 48 h treatment group was established alongside the 24 h treatment to evaluate the time-dependent anti-proliferative effect of JP. Each experiment was carried out in triplicate. The inhibition rate was calculated as follows:

Inhibition rate (%) = [1 − (A_sample_/A_control_)] × 100%

where A_sample_ and A_control_, respectively, represent the absorbance of the treatment group and the control group.

HeLa cell migration assay: HeLa cells were seeded and cultured in 6-well plates until approximately 90% confluence was achieved. Three parallel horizontal guide lines were drawn on the underside of each well using a black marker. Three vertical scratches were then made perpendicular to the guide lines, using a sterile 200 μL pipette tip, to form a grid pattern, with individual scratches spaced at least 0.5 cm apart. The wells were then gently rinsed with PBS to prevent the attachment of detached cells and debris to the scratched area. Each well then received 2 mL of serum-free medium, either without supplementation or containing JP at concentrations of 0.5, 1.0, or 2.0 mg/mL. The plate was then gently swirled to ensure even distribution and left for incubation. At 0, 24, and 48 h following scratch introduction, cell migration was observed and photographed at the intersections of the guide lines and scratches, using an inverted microscope (CKX53, Olympus, Tokyo, Japan).

Logarithmic-phase HeLa cells were treated with JP (0, 0.5, 1.0, 2.0 mg/mL) for 24 h, then trypsinized and resuspended in serum-free RPMI-1640 containing corresponding JP concentrations at a density of 10^5^ cells/mL. A Transwell migration assay was used to assess cell migration. A 200 uL aliquot of cells in serum-free medium was replated in the upper compartment of Transwell inserts, and 500 μL RPMI-1640 medium with 10% FBS in the lower compartment served as a chemoattractant. After allowing the system to incubate for 24 h, the non-migrated cells, which were still on the upper surface of the membrane, were gently removed with a cotton swab. Cells migrated to the lower surface were fixed with methanol for 15 min, dried, stained with 0.1% crystal violet for 20 min, washed with PBS to remove excess crystal violet, and then visually examined by light microscope for quantitative analysis. Hoechst 33258 staining solution staining for apoptosis: Exponentially growing HeLa cells were seeded in 96-well plates at an appropriate density and allowed to adhere for 24 h. Cells were subjected to 48 h of treatment with DMEM medium-based JP solutions (0, 0.5, 1.0, and 2.0 mg/mL) (Solarbio, Beijing, China) containing 10% BSA (Solarbio, Beijing, China). The culture medium was gently removed following treatment, and the cells were fixed with 4% paraformaldehyde for 15 min at room temperature. After this duration, the fixative was discarded, and the cells were washed twice with PBS. Subsequently, each well received 30 μL of Hoechst 33258 staining solution, followed by a 10-min incubation of the plates in the dark. The cells were then washed twice with PBS and observed under a fluorescence microscope (Axio Observer A1, Zeiss, Oberkochen, Germany).

Western Blot Analysis of Mitochondrial Apoptosis-Related Proteins: HeLa cells in the logarithmic phase were seeded in 10 cm dishes at 2 × 10^6^ cells/mL and treated with JP (0, 1.0, 2.0 mg/mL in DMEM with 10% BSA) for 48 h. RIPA lysis buffer with PMSF was employed for total protein extraction, and concentration was determined by BCA assay. Equal amounts of protein (30 μg/lane) were separated by 10–12% SDS-PAGE and transferred to PVDF membranes. The membranes were blocked with 5% non-fat milk in TBST for 1 h at room temperature before being incubated overnight at 4 °C with primary antibodies against Bax, Bcl-2, Cleaved-caspase 9, and Cleaved-caspase 3. Membranes were washed in TBST and incubated with HRP-conjugated secondary antibody for 1 h. Protein bands were visualized by ECL (ChemiDoc MP, Bio-Rad, Hercules, CA, USA) and imaged. The intensities of the bands were quantified in Image J and normalized to ACTB.

Analysis of cell cycle-related gene expression by RT-PCR: Primers corresponding to CCND1, CDK2, CDK4, CDK6, PCNA, MYC, TP53, and ACTB were designed and synthesized by Tsingke Biotechnology Co., Ltd. The details are as follows (Table 1):

HeLa cells were treated with 0, 0.1, 0.3, 0.4, 0.5, and 1.0 mg/mL JP in culture medium and then collected into centrifuge tubes. Individual centrifuge tubes then received 1 mL of TRIzol reagent, followed by repeated pipetting to ensure complete cell lysis. The mixture was placed on ice for 15 min, following which 200 µL of chloroform was added to each centrifuge tube. The resulting mixture was vigorously shaken for 15 s and incubated on ice for 3 min. The sample was then centrifuged at 12,000 rpm and 4 °C for 15 min using a high-speed refrigerated centrifuge (5810R, Eppendorf, Hamburg, Germany). From the aqueous phase, 400 µL was transferred to a new tube, mixed with 400 µL of isopropanol by gentle inversion, and left to stand at room temperature for 20 min. After centrifugation of this mixture at 12,000 rpm and 4 °C for 15 min, the supernatant was discarded. The pellet was washed with 1 mL of 75% ethanol, centrifuged again under the same conditions, and the supernatant was removed. Following air-drying at room temperature for 3 min, the pellet was resuspended in 25 µL of DEPC-treated sterile water to achieve complete dissolution of RNA.

Five microliters of the extracted RNA was transferred to an EP tube, followed by the addition of 1 µL of oligo (dT) 18 primer and 7 µL of H_2_O. Thermal denaturation was performed by incubating the reaction mixture at 70 °C for 5 min in a PCR thermal cycler (Mastercycler EP Gradient, Eppendorf, Hamburg, Germany), with the heated lid maintained at 105 °C to prevent condensation. Subsequently, 5 µL of 5× MMLV buffer, 2 µL of dNTP mixture, 0.5 µL of RNase inhibitor, 13 µL of the denatured RNA template, and 4.5 µL of H_2_O were added to the same tube. The reverse transcription reaction was carried out at 37 °C for 90 min, and following the completion of the reaction, the cDNA product was stored at −20 °C for subsequent use (Table 2).

Gel electrophoresis of amplified PCR products was performed on 1.5% agarose gels at 110 V for 25 min and was then visualized with a gel imaging system (GenoSens 2250, Qinxiang Scientific Instrument Co., Ltd., Shanghai, China). The amplified fragments of the CCND1, CDK2, CDK4, CDK6, PCNA, MYC, TP53, and ACTB (as an internal control) were analyzed by densitometric analysis using Image J software. The signal intensities of each of the target genes were then normalized to the signal intensity of ACTB to determine relative transcript abundance. Data were analyzed and processed by GraphPad Prism version 6.01.

Preparation of JP-loaded thermosensitive hydrogel: 1.7 g of gelatin was immersed in 20 mL of distilled water and soaked for 3–4 h to complete water absorption and sufficient swelling. Next, 1.0 g of chitosan powder was added into 50 mL aqueous solution containing 10 wt% citric acid, followed by continuous magnetic stirring (RCT Basic, IKA-Werke, Staufen im Breisgau, Germany) until a homogeneous, transparent, bubble-free viscous system was acquired. In addition, 1.8 g of F127 powder was dispersed in 30 mL deionized water and kept stationary at room temperature for 2–3 h for swelling. After finishing the pretreatment of the three components, the fully swollen gelatin was firstly incorporated into the chitosan-citric acid solution, and the preformulated F127 aqueous solution was then supplemented under continuous stirring. During homogenization of the mixed system, 1 g of JP powder was incorporated and stirred until uniform dispersion. Afterwards, 6.25 mL of glutaraldehyde solution (25 wt%) was added dropwise slowly to initiate the crosslinking and curing reaction of the whole system. For clarity, the JP group is also denoted as JP and the JP-loaded hydrogel group as JP-H. Sol-gel behavior was evaluated by vial inversion at different temperatures to ensure the injectability of the formulation at lower temperatures and stable gel formation near 37 °C. The interaction among the pigment and hydrogel matrix components was assessed via FTIR analysis (Nicolet iS50, Thermo Scientific, Madison, WI, USA). Visual appearance, moldability, and in situ gelation behavior were documented photographically.

Antibacterial assay of JP-H: The hydrogel system was evaluated for its antibacterial activity against *E. coli* and *S. aureus*. PBS, blank hydrogel, and free JP and JP-H were incubated with bacterial suspensions. Antibacterial performance was assessed by colony formation on agar plates, whereas bacterial morphology was observed via scanning electron microscopy (SU8010, Hitachi High-Tech, Tokyo, Japan), wherein bacterial inhibition or bactericidal action was assessed in terms of reduced colony numbers and altered bacterial morphology.

Time-kill curve assay: The time-dependent antibacterial activity of free JP, blank hydrogel, and JP-H was evaluated against *E. coli* and *S. aureus*. Bacterial suspensions were adjusted to approximately 10^6^ CFU/mL in PBS and incubated with the respective samples at 37 °C under shaking. At predetermined time intervals (0, 1, 2, 4, 6, 8, 12, and 24 h), aliquots were withdrawn, serially diluted with sterile PBS, and plated on Luria-Bertani (LB) agar plates. Colony-forming units (CFU) were counted after 24 h of incubation at 37 °C. Viable bacterial counts were expressed as log_10_CFU/mL and plotted against time to construct time-kill curves. All experiments were performed in triplicate.

In vitro antitumor activity of JP-H: HeLa cells were used to evaluate the antitumor activity of the JP-loaded hydrogel. For this purpose, cells were treated with PBS, blank hydrogel, free JP, and JP-H. The cell migratory capacity was determined by a wound-healing assay, while the cell viability was assessed with a live/dead staining using FITC and PI fluorescence channels and a Hoechst fluorescence channel (Axio Observer A1, Zeiss, Oberkochen, Germany). The therapeutic efficacy of the JP-loaded hydrogel was then compared to the free JP formulation to assess whether the hydrogel was more effective at improving the retention of JP and tumor eradication at the diseased site.

Annexin V-FITC/PI double staining flow cytometry assay: HeLa cells were seeded into 6-well culture plates and incubated overnight to reach adherent growth. The cells were divided into four groups: Control, free JP, blank thermosensitive hydrogel, and JP-H. After 24 h of co-culture with corresponding samples, the culture medium was discarded, and cells were digested with trypsin, collected and washed twice with PBS.

Cell pellets were resuspended in 100 μL of Annexin V-FITC binding buffer. Subsequently, 5 μL Annexin V-FITC and 5 μL PI staining solution were added sequentially, mixed gently, and incubated in the dark at room temperature for 15 min. After incubation, another 400 μL binding buffer was supplemented, and the samples were immediately analyzed by flow cytometry (FACSCalibur; BD Biosciences, San Jose, CA, USA). The fluorescence signals of Annexin V-FITC and PI were recorded to distinguish live cells, early apoptotic cells, late apoptotic cells and necrotic cells, and the proportion of apoptotic cells in each group was quantitatively calculated.

Statistical analysis: All data were expressed as the mean ± SD. Data were analyzed statistically by SPSS 11.5. ChemBioDraw Ultra 12.0 was used to draw chemical structures. * *p* < 0.05 was regarded as significant and ** *p* < 0.01 indicated a high level of statistical significance.

## Figures and Tables

**Table 1:** Sequences of primers used for RT-PCR analysis of cell cycle-related gene expression.

Gene	Orientation and Sequences
*CCND1*	Forward: 5′TTCGTGGCCTCTAAGATGAAG 3′
	Reverse: 5′GTAGGACAGGAAGTTGTTGGG 3′
*CDK2*	Forward: 5′TCAAGCTAGCAGACTTTGGAC 3′
	Reverse: 5′ACTTGGCTTGTAATCAGGCAT 3′
*CDK4*	Forward: 5′AATTGCATCGTTCACCGAGAT 3′
	Reverse: 5′CAGCCCAATCAGGTCAAAGAT 3′
*CDK6*	Forward: 5′CAGTGTCACGAACAGACAGAG 3′
	Reverse: 5′GGTTAGAGCCATCTGGAAACT 3′
*PCNA*	Forward: 5′TTGGCGCTAGTATTTGAAGCA 3′
	Reverse: 5′CAGGTACCTCAGTGCAAAAGT 3′
*MYC*	Forward: 5′CTTCTCTCCGTCCTCGGATTC 3′
	Reverse: 5′GGATAGTCCTTCCGAGTGGAG 3′
*TP53*	Forward: 5′CCTGTCATCTTCTGTCCCTTC3′
	Reverse: 5′CTCGGATAAGATGCTGAGGAG3′
*ACTB*	Forward: 5′TCTACAATGAGCTGCGTGTGG3′
	Reverse: 5′GAGGTAGTCAGTCAGGTCCCG3′

**Table 2:** PCR reaction components.

Component	Volume (µL)
2 × F8 PCR Master MIX	10
Forward Primer (10 μM)	0.5
Reverse Primer (10 μM)	0.5
cDNA Template	0.8
DMSO	5
Nuclease-free Water	To 20

## Data Availability

The data presented in this study are available upon request from the corresponding author. The data are not publicly available due to ethical reasons.

## Supplementary Materials

The following supporting information can be downloaded at: https://www.mdpi.com/article/10.3390/gels12090788/s1, Supplementary Figure S1: HPLC chromatogram of the authentic delphinidin-3-O-rutinoside standard.
