# Supplementary material for: Jujube Peel Pigment-Loaded Thermosensitive Hydrogel with In Vitro Pro-Apoptotic and Antibacterial Activities
Source: Gels. 2026 Sep 1;12(9):788. doi: 10.3390/gels12090788 (PMC13606395; doi:10.3390/gels12090788)
Supplement: Supplementary file 1 [file gels-12-00788-s001.zip › gels-4478003-supplementary.pdf]

Supplementary Figure:

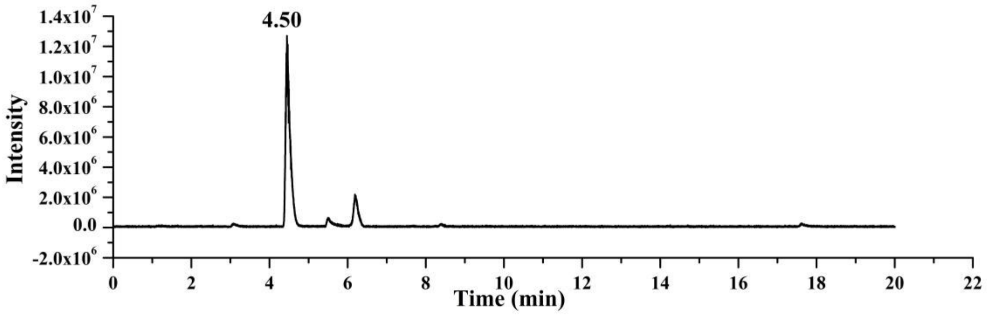

Supplementary Figure S1: HPLC chromatogram of the authentic delphinidin-3-O-rutinoside standard. The standard was analyzed under the same chromatographic conditions as described for the sample in Figure 2c. A single sharp peak was eluted at a retention time of 4.50 min.
